# A Case of a Short Stature Patient With Marfan Syndrome: A Tale of Wide Mediastinum

**DOI:** 10.7759/cureus.76583

**Published:** 2024-12-29

**Authors:** Yasser Hegazy, Alshaimaa Abdallah, Ahmed Salem, Ali Assaker, Ahmed Elmogy

**Affiliations:** 1 Internal Medicine, Icahn School of Medicine at Mount Sinai, Queens Hospital Center, New York, USA; 2 Radiology, Cairo University, Cairo, EGY; 3 Internal Medicine, CarePoint Health Bayonne Medical Center, Bayonne, USA

**Keywords:** aortic aneurysm, aortic valve insufficiency, ascending aortic aneurysm, marfan syndrome, severe aortic regurgitation

## Abstract

Marfan syndrome (MFS), an inherited connective tissue disorder, is caused by a mutation in the FBN1 gene. MFS is characterized by complex manifestations involving musculoskeletal, cardiovascular, and ocular systems. The usual presentation for suspecting diagnosis in an individual with aortic root disease is tall stature in addition to other features that fulfill Ghent criteria. On the other hand, we report a case of a 30-year-old male patient with markedly dilated aortic root fulfilling diagnoses of MFS despite being of relatively short stature.

## Introduction

Marfan syndrome (MFS) is a multisystemic connective tissue disorder with autosomal dominant inheritance, affecting approximately 1 in 5,000 individuals, though this may be an underestimation. The condition occurs across all races and geographic regions, displaying complete penetrance but variable expression and age-related onset. Around 25% of cases arise from de novo mutations, without any family history [1]. MFS is characterized by pleiotropy, with a wide range of manifestations across different organ systems, including the ocular, cardiovascular, and musculoskeletal systems [2]. Clinical severity varies significantly, and diagnosis primarily relies on meeting clinical criteria established by the revised Ghent nosology [3]. The mere presence of aortic root dilatation Z score ≥ 2 and systemic score ≥ 7 points is enough for MFS diagnosis. Individuals with MFS typically exhibit greater height than expected for their genetic background, with average final heights of 191.3 ± 9 cm in men and 175.4 ± 8.2 cm in women [4]. In this report, we present a challenging case with an extremely dilated aortic root that was diagnosed as MFS based on the presence of a dilated aortic root and fulfilling systemic score despite being of low normal height compared to the average height reported in the Marfan population. It is peculiar given the fact that the middle-aged patient with low normal height presented with a hugely dilated ascending aorta and no risk factors for weak mesenchyme as well as the absence of family history. Typically, clinicians who see cases of this hugely dilated aorta would expect clues from family history and gross physical examination. In this particular case, if it was not for the holistic physical (including particular detailed physical findings pertinent to MFS; wrist/thumb signs, elbow extension angle, and hyperextensibility of knee joint), the appropriate underlying diagnosis would have been easily missed. It also emphasized that it is not uncommon for Marfan to be de novo in the absence of family history. In brief, MFS should be suspected in any case of abnormally dilated aortic root and active assessment for other signs of MSF should be sought with family screening for first-degree relatives even if no family history is present.

## Case presentation

**Figure 1:**
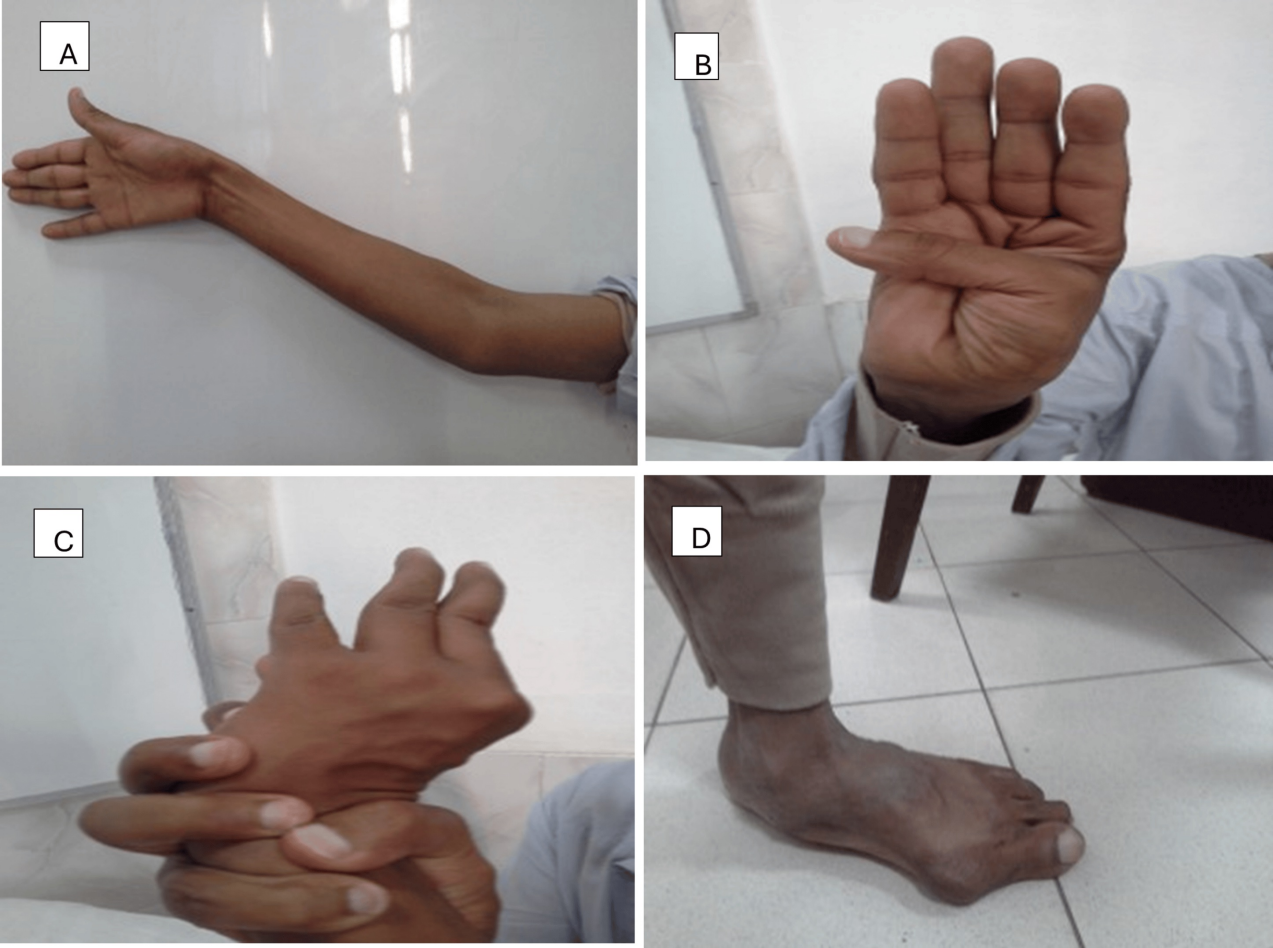
(A) Reduced elbow extension, (B) Thumb sign, (C) Wrist sign, and (D) Pes planus

**Figure 2:**
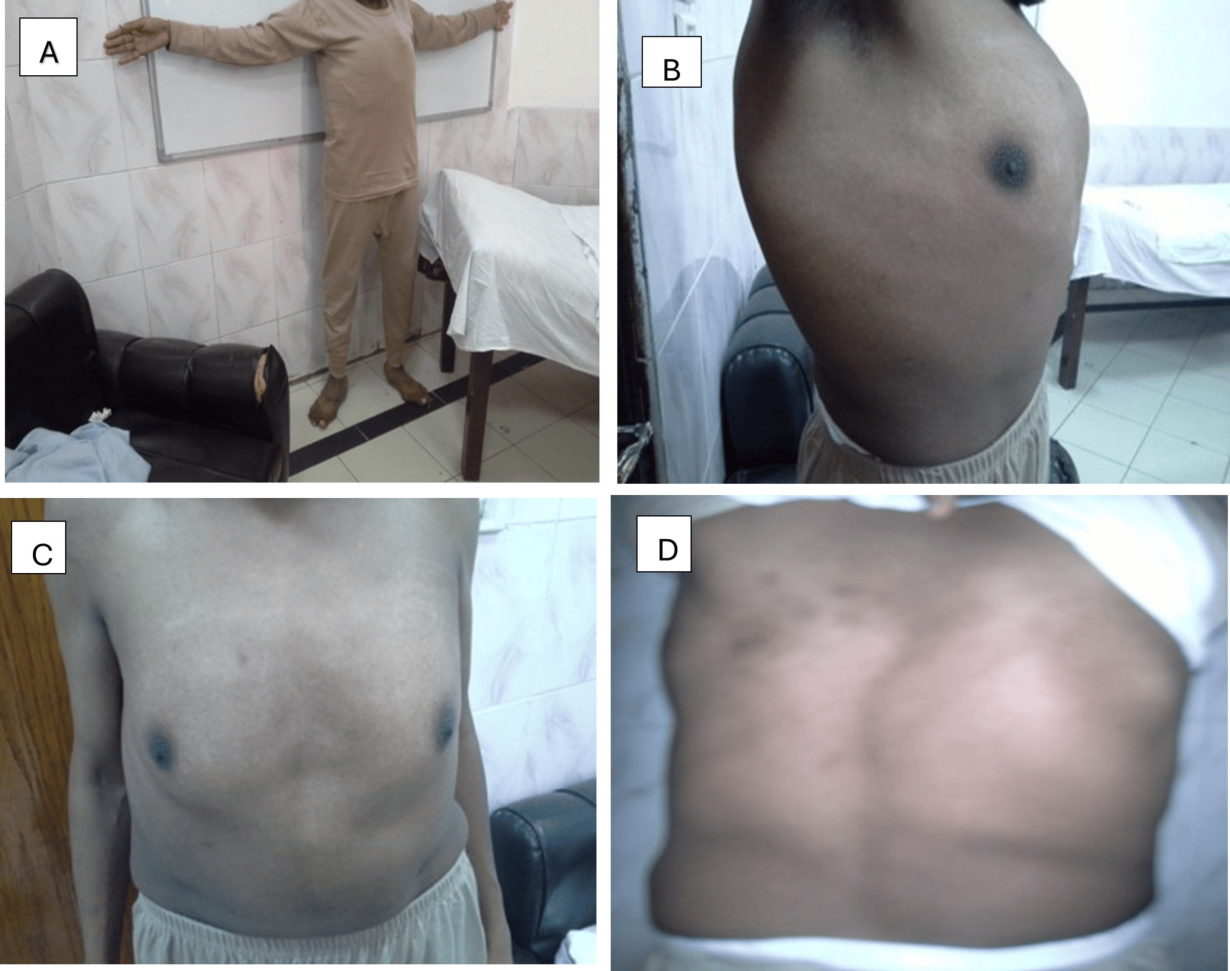
(A) Reduced US/LS and increased arm span/height, (B, C) Chest asymmetry, and (D) Scoliosis US: Upper segment; LS: lower segment

**Figure 3:**
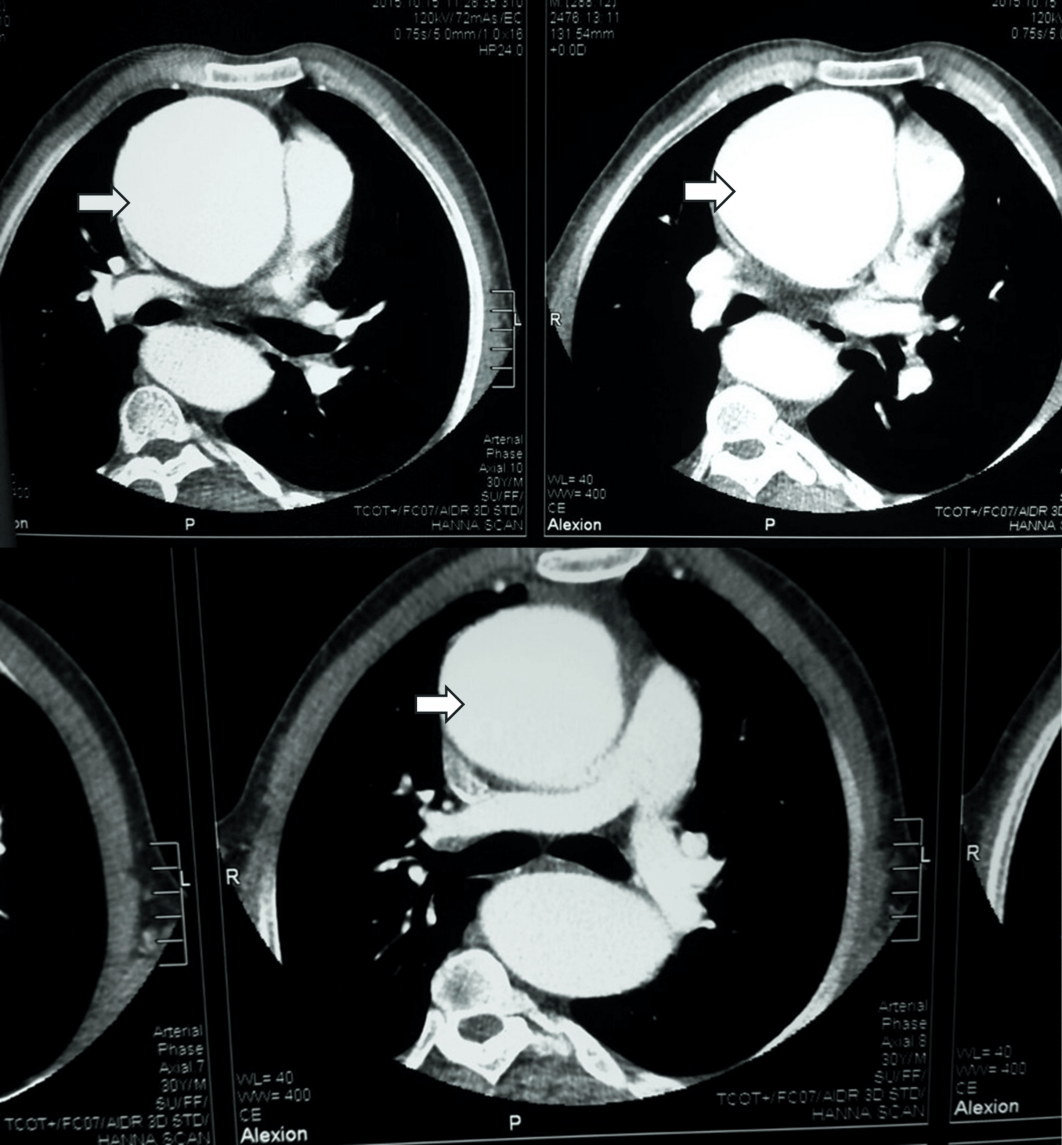
Arrows indicate large ascending aortic aneurysm in CT aortography

**Figure 4:**
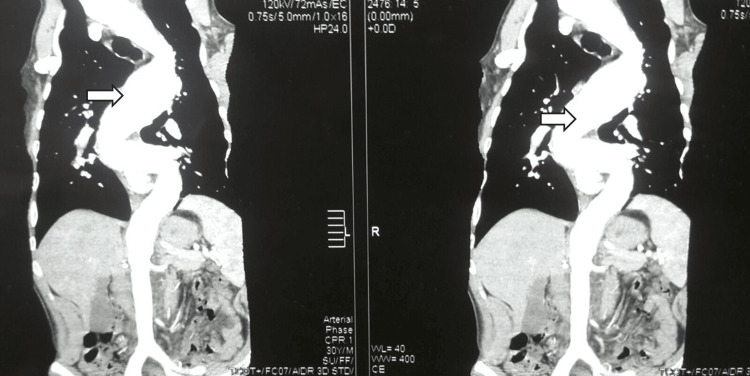
Arrows indicate elongation and tortuosity of thoracic aorta in CT aortography

A 30-year-old male patient, heavy “hookah” smoker with no significant past medical history and history of varicocelectomy at the age of 25, presented with gradual progressive shortness of breath on minimal exertion of six weeks duration. This was associated with occasional exertional palpitations and dizziness. The patient has a family history of sudden cardiac death (SCD) (mother and sister, at the age of 37 and 32, respectively). On presentation, the patient was alert and oriented to time, place, and person. Blood pressure was 130/40, equal on both sides and 160/50 in both lower limbs (Hill’s phenomenon). Pulse is 80 bpm, regular with big pulse volume with characteristic pulsus bisferens. Body parameters were significant for height 169 cm (low-normal height for average Egyptian population), span 178 cm, (upper segment US/ lower segment LS 0.69), weight 69 Kg, and BMI 24. Head and neck examination revealed high arched palate with visible pulsations at the neck “Corrigan’s sign”, and systolic tracheal tug “Oliver’s sign”. The musculoskeletal examination was significant for an asymmetrical chest with increased anteroposterior diameter in relation to the transverse diameter, thoracolumbar scoliosis, thumb sign, Steinberg wrist sign, limited elbow extension (≤170˚), hyperextensibility of both knee joints, plain flat feet (pes planus), and hallux valgus of left foot (Figures 1, 2). Cardiovascular examination showed a dilated left ventricle with a shift of apex to the left 6th intercostal space, hyperdynamic apex with systolic bulge, and right and left parasternal and pulmonary pulsations. Cardiac auscultation revealed normal S1, accentuated pulmonary component of S2, S3 over the mitral area, the early diastolic murmur of aortic regurgitation on the 1st and 2nd aorta area propagating along the parasternal area, of grade IV intensity and mid-diastolic rumbling murmur at the apex (Austin Flint murmur). The echocardiogram showed severely dilated left ventricular internal dimensions (LVED= 8.4, LVES= 6.1) with low normal global contractility (EF=51% by Simpsons’ method), dilated ascending aorta (dilated annulus measuring 4.1 cm, widest coronary sinus to coronary sinus measurement is 8.3 cm, above sinuses it measures 9.1 cm at its widest diameter and effacement of sino-tubular junction), and tri-leaflet aortic valve with severe aortic incompetence. CT aortography revealed ascending aortic aneurysm (9 cm from the root measuring 10 cm) aortic arch and thoracic aorta elongation, tortuosity, and severe kinks involving the roots of three major branches of the aorta (Figures 3, 4). In addition, the segmental area of thoracic aorta dilatation was noted (measuring 5.5 cm) distal to the origin of the left subclavian artery running for 12 cm with normal abdominal aorta and pulmonary artery. Therefore, this person fulfilled the diagnosis of MFS despite being of short stature in relation to the Marfan population. The diagnosis was based on Aortic root dilatation Z score of 19.3 (>2) and systemic features score of 8 (wrist and thumb sign; 3 points, chest asymmetry; 1 point, reduced elbow extension; 1 point, scoliosis; 1 point, pes planus; 1 point, reduced US/ LS and increased arm span/height; 1 point) (Figures 1, 2) which resolved the mystery of severe aortic root dilatation. The history of SCD in his family further confirms the diagnosis; however, due to the lack of documentation, no clear etiology was discovered. The patient was started on bisoprolol 2.5 mg tablet once daily for heart rate control, lisinopril 20 mg tablet once daily for afterload reduction in setting of severe AR, and frusemide 20 mg tablet two times daily for control of heart failure symptoms. While inpatient, he was directly referred for surgery. The Bentall procedure with a bioprosthetic aortic valve was successfully performed, and the patient was discharged after an uneventful post-operative hospital stay. On interval follow-ups at two weeks, one month, three months, six months, and yearly for two successive years, the patient reported improvement of heart failure symptoms. Echocardiograms on follow-ups at two weeks, three months, and one year showed gradual improvement of LV dimensions with the last EF reported at one year 65%. In addition, screening of the first-degree family relatives revealed that they were free of systemic features of MFS as well as the absence of aortic root dilatation.

## Discussion

MFS is primarily inherited through an autosomal dominant pattern, with the majority of cases linked to mutations in the FBN1 gene, which encodes fibrillin-1, a key protein in connective tissue. Although most individuals with MFS inherit the condition from an affected parent, approximately 25% of cases arise from de novo mutations [5]. MFS is characterized by connective tissue abnormalities, including ocular lens dislocation and skeletal deformities; however, nearly all adults with the syndrome develop cardiovascular abnormalities, which account for the majority of premature deaths in this population [6]. The diagnosis of MFS was based on clinical criteria outlined in the Ghent nosology (1996) [3]. In 2010, the Ghent nosology criteria were updated (Table 1) to emphasize two primary indicators of MFS: aortic root aneurysm or dissection and ectopia lentis. When other findings of MFS are absent, the presence of both ectopia lentis and aortic root enlargement or dissection is sufficient for diagnosis. Additional cardiovascular and ocular features, as well as manifestations in other systems, such as the skeletal, dural, dermatologic, and pulmonary systems, are included in a “systemic score” (Table 2), which aids in diagnosis in cases where aortic disease is present without ectopia lentis [7]. In the absence of family history and genetic testing, the diagnosis of MFS in our patient was confirmed by an aortic root dilation Z-score of 19.34, along with a systemic feature score of 8. The aortic root Z score was developed to outline the natural distribution of normal aortic root dimension ranges in the normal population. Scores equal to or more than 2 are considered abnormally high, hence dilated. In addition, the patient had systemic features score >7 (wrist and thumb sign; 3, chest asymmetry 1, reduced elbow extension 1, scoliosis 1, pes planus 1, reduced US/ LS, and increased arm span/height 1 with a total of 8)

**Table 1:** Revised Ghent (2010 Ghent nosology) criteria Ao: Aortic root diameter at the sinuses of Valsalva above-indicated score Z-score or Aortic dissection; EL: Ectopia Lentis; ELS: Ectopia lentis syndrome; FBN1: Fibrillin-1 mutation; FH: Family history; MASS: Mitral valve prolapse, borderline (<2) Z-score aortic root dilatation, striae, skeletal findings phenotype; MFS: Marfan syndrome; MVP: Mitral valve prolapse; MVPS: Mitral valve prolapse syndrome; Syst: Systemic score; Z: Z score Source: [7]

Revised Ghent (2010 Ghent nosology) criteria
In the absence of family history:
Ao (Z ≥2) AND EL = MFS
Ao (Z ≥2) AND FBN1 = MFS
Ao (Z ≥2) AND Syst ≥ 7 points = MFS
EL and FBN1 with known Ao = MFS
EL with or without Syst AND with an FBN1 not known with Ao or no FBN1 = ELS Ao (Z < 2) AND Syst (≥ 5 with at least one skeletal feature ) without EL =MASS Ao (Z <2) AND Syst ( < 5 ) without EL = MVPS
In the presence of family history:
EL AND FH of MFS = MFS
Syst (≥ 7 points) AND FH of MFS = MFS
Ao ( ≥2 above 20 years , ≥3 below 20 years ) AND FH of MFS = MFS

**Table 2:** Scoring of systemic features (2010 Ghent nosology) Source: [7]

Feature	Value
Wrist AND thumb sign	3
Wrist OR thumb sign	1
Pectus Carinatum deformity	2
Pectus excavatum or chest asymmetry	1
Hindfoot deformity	2
Plain flat foot (pes planus)	1
Pneumothorax	2
Dural ectasia	2
Protrusio acetabuli	2
Reduced upper segment/lower segment and increased arm span/height	1
Scoliosis or thoracolumbar kyphosis	1
Reduced elbow extension	1
Three of five facial features (dolichocephaly, enophthalmos, down slanting palpebral fissures, malar hypoplasia, retrognathia)	1
Skin striae	1
Myopia > 3 diopters	1
Mitral valve prolapsed (all types)	1

In a study of U.S. individuals with the Marfan population, Gurkan Erkula analyzed growth patterns and developed growth charts for individuals with MFS [4]. Longitudinal height and weight measurements were collected from 180 patients with a clinical diagnosis of MFS. Based on these data, growth and growth velocity charts were created for both men and women. The mean final height was 191.3 ± 9 cm for men and 175.4 ± 8.2 cm for women. The mean birth weight was 3.51 ± 0.74 kg for men and 3.48 ± 0.68 kg for women [4]. In a separate Dutch study, the mean final height of individuals with MFS from the Netherlands was compared to results from similar studies conducted in the U.S., France, and Korea [4,8-10]. The findings are presented in Table 3.

**Table 3:** Comparison of studies of linear growth in male individuals with Marfan syndrome MFS: Marfan syndrome; SD: standard deviation; HSDS: height standard deviation score

Study population	Study sample	Molecularly confirmed MFS	Mean final height ± SD of individuals with MFS	Mean final height ± SD of the reference population	MFS mean final height expressed as HSDS of the reference population ± SD (p-value)
United States, 2002 (Erkula et al. [4])	99 men	NO	191.3 ± 9 cm	176.7 ± 7.1 cm	2.1 ± 1.3 (0.0406)
Korea, 2015 (Kwun et al. [8])	187 men	NO	191.5 ± 5.3 cm	173.4 ± 5.6 cm	3.2 ± 0.9 (<0.0001)
France, 2018 (Benoist et al. [9])	134 men	YES	191.2 ± 8.4 cm	175.2 ± 7.6 cm	2.1 ± 1.1 (0.0053)
The Netherlands, 2023 (Lauffer et al. [10])	210 men	YES	195.3 ± 7.3 cm	182.4 ± 7.3 cm	1.8 ± 1.0

This case appears to be unique, as no similar instances have been documented in the literature, whether systematic review studies or Marfan syndrome registries. The distinctiveness arises from the patient's relatively short stature (169 cm), an uncommon trait among individuals with MFS. Among the previously mentioned studies across different populations, this patient's documented height is significantly lower than the mean average of the aforementioned populations. Furthermore, the patient exhibited a significantly enlarged aortic root aneurysm measuring 10 cm in diameter (with Z score >19.34), accompanied by severe aortic regurgitation. Management of aortic dilatation in MFS patients generally involves routine echocardiographic monitoring, beta-blocker therapy, exercise limitations, and surgical intervention. Beta-blockers are advised for all MFS patients, particularly younger individuals and those with an aortic diameter exceeding 40 mm [11]. For patients who cannot tolerate beta-blockers or where they are contraindicated, alternative therapies that reduce ejection impulse, such as angiotensin-converting enzyme (ACE) inhibitors, may be considered, although clinical trial evidence supporting their efficacy in MFS is currently lacking with further observation studies needed to give more solid clinical evidence for therapy. In our case, the patient received both beta-blockers and ACE inhibitors briefly before scheduled surgery for heart rate control and afterload reduction and both are also pillars for heart failure management. Oral Furosemide was also prescribed for symptomatic treatment of heart failure.

In addition, according to the latest guidelines from the American College of Cardiology, elective aortic root and aortic valve replacement is recommended for our patient [12] both for the severe AR as well as the dilated aortic root. For isolated dilated aortic root, prophylactic surgery is recommended for adults when the diameter of the sinus of Valsalva surpasses 50 mm even in the absence of MFS, with lower cut-offs designated for different connective tissue disorders including MFS. Additional considerations, such as the rate of aortic dilatation per year, planning for pregnancy, and family history of aortic dissection, may also influence the decision for surgical intervention [13]. Potential risks of delaying surgery (including aortic dissection, worsening heart failure, cardiogenic shock, and frequent hospitalization) in patients with dilated aortic root and connective tissue disorder are avoided by early surgery. In addition, the presence of severe AR in this case was an even more solid indication for early surgery even in the absence of aortic root dilatation.

## Conclusions

This case highlights an unusual presentation of MFS in a patient with relatively short stature, challenging the typical association of MFS with tall stature. This could pave the way for future observational studies across different populations with systemic features or positive genetic testing, reporting the natural distribution of height. The patient's diagnosis was established based on significant aortic root dilation and systemic features. Clinicians should maintain a high index of suspicion in any case of dilated aortic root and perform holistic physical examination bearing in mind systemic features listed in the revised Ghent criteria. Clinicians should also be aware of the Z-score calculators for the diagnosis of dilated aortic root which are widely available on vast cell phone applications as well as on the internet. This case also reinforces the critical role of genetic testing, especially in unlimited resource settings, in diagnosing MFS in atypical phenotypic variants. This will aid in identifying and early management of atypical cases of MFS, especially in patients with family histories of SCD. Early intervention remains crucial to improving patient outcomes in such presentations.
